# Investigating the impact of outpatient services on length of stay: an easily interpretable approach

**DOI:** 10.3389/frai.2026.1746547

**Published:** 2026-03-25

**Authors:** Roberta Bellini, Simone Garau, Riccardo Gualiumi, Giorgio Leonardi, Stefania Montani, Manuel Striani, Cristian Zanelli

**Affiliations:** 1 Laboratorio Integrato di Intelligenza Artificiale e Informatica in Medicina DAIRI, Azienda Ospedaliera SS. Antonio e Biagio e Cesare Arrigo, Alessandria, Italy; 2 DISIT, Computer Science Institute, University of Piemonte Orientale, Alessandria, Italy

**Keywords:** explainability, length of stay, LLM, process model discovery, process trace classification

## Abstract

Limiting hospital length of stay (LOS) can prevent patient complications and reduce costs. The identification of activities that could impact LOS, and which may be attributable to outpatient services (OSs - i.e., diagnostic exams or specialist consultations provided by external wards), can be very useful for hospital administrators, and help optimize healthcare processes. In this work, we introduce TEXLoS, a tool able to study the association of OS activities with LOS. The problem is afforded in a Business Process Management perspective, allowing the integration and the adoption of state of the art techniques for trace classification and process model discovery. The focus of TEXLoS output is on explainability, which is the key to the adoption of the tool in practice. In the paper, we present the main steps of the TEXLoS architecture, and the results of its application to the data of *Azienda Ospedaliera SS. Antonio e Biagio e Cesare Arrigo* in Alessandria, Italy, where a balanced accuracy of 93% has been reached, along with a Matthews Correlation Coefficient of 0.68, confirming the high performance in classification. Interpretability of the provided output has also been successfully validated by a group of end user.

## 1 Introduction

Length of stay (LOS) is a metric which quantifies the amount of time a patient remains in the hospital, starting from admission and ending at discharge (Gokhale et al., 2023). Prolonged hospital stays can result in negative outcomes, including increased risk of hospital-acquired infections or other complications, and elevated costs for both patients and the healthcare system (Bueno et al., 2010; Rotter et al., 2010). Frequently, delays in discharge are caused by avoidable waiting times, inefficient decision-making, or shortcomings in care and service coordination (Rojas-García et al., 2018).

In Italy, patients are admitted to the ward where their primary procedure or treatment will take place. However, during their hospital stay, they often require additional services, such as diagnostic tests or specialist consultations, which are performed by other wards. These are known as Outpatient Services provided during hospitalization (OSs henceforth). For instance, before femur fracture surgery, a patient will typically have X-rays in the radiology ward; after the intervention, s/he will require a physiatric consultation to establish rehabilitation steps. The admitting ward must request these OSs and then wait for the necessary external resources to become available, which can sometimes lead to delays.

In this paper, we aim at investigating whether OSs have an impact on LOS. The approach we propose is characterized by two choices:

1. We afford the problem in a Business Process Management (BPM) perspective: this allows us to take advantage of a large amount of techniques described in the literature, for analyzing the input situation, and for studying possible solutions;
2. We focus on the provision of explainable tools, where the end user can easily understand the key elements that were considered during the analysis, and thus be guided in the choice of a possible improvement strategy.

As regards item (1), it is worth mentioning that an analysis of LOS directly working on tabular data has been recently proposed (Andric and Dragoni, 2025); on the other hand, in this paper we propose to transform the hospital information system tabular data into process traces (van der Aalst, 2016), i.e., sequences of timestamped OS activities carried out on the various patients within the healthcare organization. This allows us to fully exploit the advantage of the integration with BPM tools. BPM is indeed a lively area of research, which has made available a large set of methods to discover, model, analyze and optimize business processes (van der Aalst, 2016). These methods are available both within open source frameworks and in commercial tools, and are typically characterized by user-friendly interfaces, which enable users to understand all the steps of the process life cycle, in a transparent way. Moreover, such suites offer an integrated environment, where users can easily find instruments to define/mine the process, but also to monitor it over time, and to make improvements, if necessary. Several BPM solutions are being exploited in medical applications as well, provided that the peculiarities of these domains have been addressed (Rojas et al., 2016).

As regards item (2), the output of automated data analysis solutions, and in particular of machine/deep learning ones, which represent nowadays the state of the art also on BPM data, needs to be made transparent to non-technical end users. If this issue is not addressed, despite the quality of the model performance, the data analysis tool will not be adopted in practice, since physicians or hospital administrators will neither be able to inspect the logic behind the suggestions, nor to understand what input features the model focused on when reaching its conclusions. In this paper, therefore, we propose to study the impact of OSs on LOS by means of a deep learning approach, with a strong focus on the need for “opening the black box." To this end, we propose to rely on Tranformers (Vaswani et al., 2017) (and specifically on Large Language Models), which are progressively becoming the state of the art in deep learning classification and prediction, and which can support transparency, both qualitatively (thanks to the attention map) and quantitatively [through, e.g., the Integrated Gradients technique (Sundararajan et al., 2017)].

In particular, we study the impact of OS activities by means of predictive classification: if OS traces correctly classify along the dimension of LOS (long vs. short), it can indicate that OSs are relevant to determine hospital stay duration. We also investigate which OS activities have the larger effect.

In order to provide further insight and transparent justification of the outcome, and to support hospital administrators in the reduction of LOS, we also adopt process model discovery techniques, to learn process models (possibly stratified by patient groups), in search of bottlenecks and other useful information for organizational improvements.

All the facilities mentioned above are made available to the end user through our tool, called Trace EXplanations for Length-of-Stay (TEXLoS), which has been validated by a group of users at the *Azienda Ospedaliera SS. Antonio e Biagio e Cesare Arrigo* in Alessandria, Italy.

The paper is organized as follows: in section 2 we describe related work; in section 3 we present the technical details of TEXLoS; in section 4 we show experimental results, while in section 5 we provide our concluding remarks.

## 2 Related work

Process traces are a valuable source of information and have been utilized to support a variety of tasks in process mining (van der Aalst, 2016), also within the healthcare sector (Munoz-Gama et al., 2022; Aversano et al., 2025). Trace classification and prediction, in particular, are nowadays addressed mainly through deep learning approaches.

Several deep learning models have been introduced to predict the next activity in a trace, such as Autoencoders (Hinton and Salakhutdinov, 2006) (in Mehdiyev et al., 2017), Convolutional Neural Networks (CNNs) (Alom et al., 2019) (in Mauro et al., 2019), and Long Short-Term Memory (LSTM) recurrent networks (Hochreiter and Schmidhuber, 1997) (in Evermann et al., 2017; Tax et al., 2018; Camargo et al., 2019; Tax et al., 2017). The most performing approaches, however, are those in (Philipp et al. 2020) and (Bukhsh et al. 2021), which utilize Transformer architectures—models that replace recurrence with the attention mechanisms (Vaswani et al., 2017). Transformers are increasingly recognized as the state of the art not only in Natural Language Processing and time series forecasting, but also in next activity prediction for predictive process monitoring (e.g., Donadello et al., 2023). These methods specifically employ multi-head attention, which performs attention across multiple segments of the trace in parallel. The use of Transformers has also extended to classification tasks, where the target class is modeled as a special token to be predicted. The attention mechanism repeatedly transforms representation vectors of the input features, incorporating more and more semantic relationships between them. By means of the attention map, the model can visually highlight the input portions that were deemed as the most important for classification. Moreover, gradient-based techniques such as Integrated Gradients (IG) (Sundararajan et al., 2017) can quantify which elements in an input sequence have significant impact on the model's output.

Very recently, Large Language Models (LLMs), which are architectures based on a Transformer, trained on huge text data, are being proposed in BPM as well [see e.g., the survey in (Berti et al. 2024)]. The work in (Pyrih et al. 2025), for instance, relies on LLMs to support semantic-aware process mining, and adopts instruction-tuning to reach satisfactory results in next activity prediction. The work in (Pasquadibisceglie et al. 2025) introduces a BPM method, called LEGOLAS, that uses an LLM to predict hospital admission for emergency patients, in an explainable way. LEGOLAS uses a story telling approach to describe patient traces, to remove artifacts.

Besides trace classification/prediction, in our approach we also adopt process model discovery (van der Aalst, 2016) techniques. Process model discovery focuses on generating a process model from the logged traces. Probably the simplest formalism to represent a process model is the Directly Follows (DF) model, a directed graph where nodes denote activities in the traces and edges capture their execution order. A DF model can be provided in output by mining algorithms, such as Directly Follows visual Miner (DFvM) (Leemans et al., 2019). DFvM first discovers the model from the available traces, and then enhances it with frequency information (i.e., how many instances - e.g., clients, patients—have crossed a given model edge between two activities), and temporal information (i.e., how long it takes, on average, to start an activity after the completion of the previous one, on a given path). A process model decorated with such information provides a useful view of how the organization is working, in a simple and transparent way.

These algorithms are available both within open source frameworks such as ProM (van Dongen et al., 2005) and Visual Miner^1^, and in commercial tools such as DISCO^2^. Interestingly, it is easy to integrate process mining algorithms into other tools (as it is needed in our case), through the open-source Python library PM4Py (Berti et al., 2023) or through the ProM Quick Visualiser^3^.

Process discovery techniques have demonstrated effectiveness across various domains, including business and manufacturing processes, but also medical ones (Rojas et al., 2016).

In our tool, we combine the most recent advances of the areas of research sketched above. Details will be provided in section 3.

## 3 Method

In this section, we first illustrate the architecture of our TEXLoS tool; we then provide further details of its main steps.

**Figure 1:**
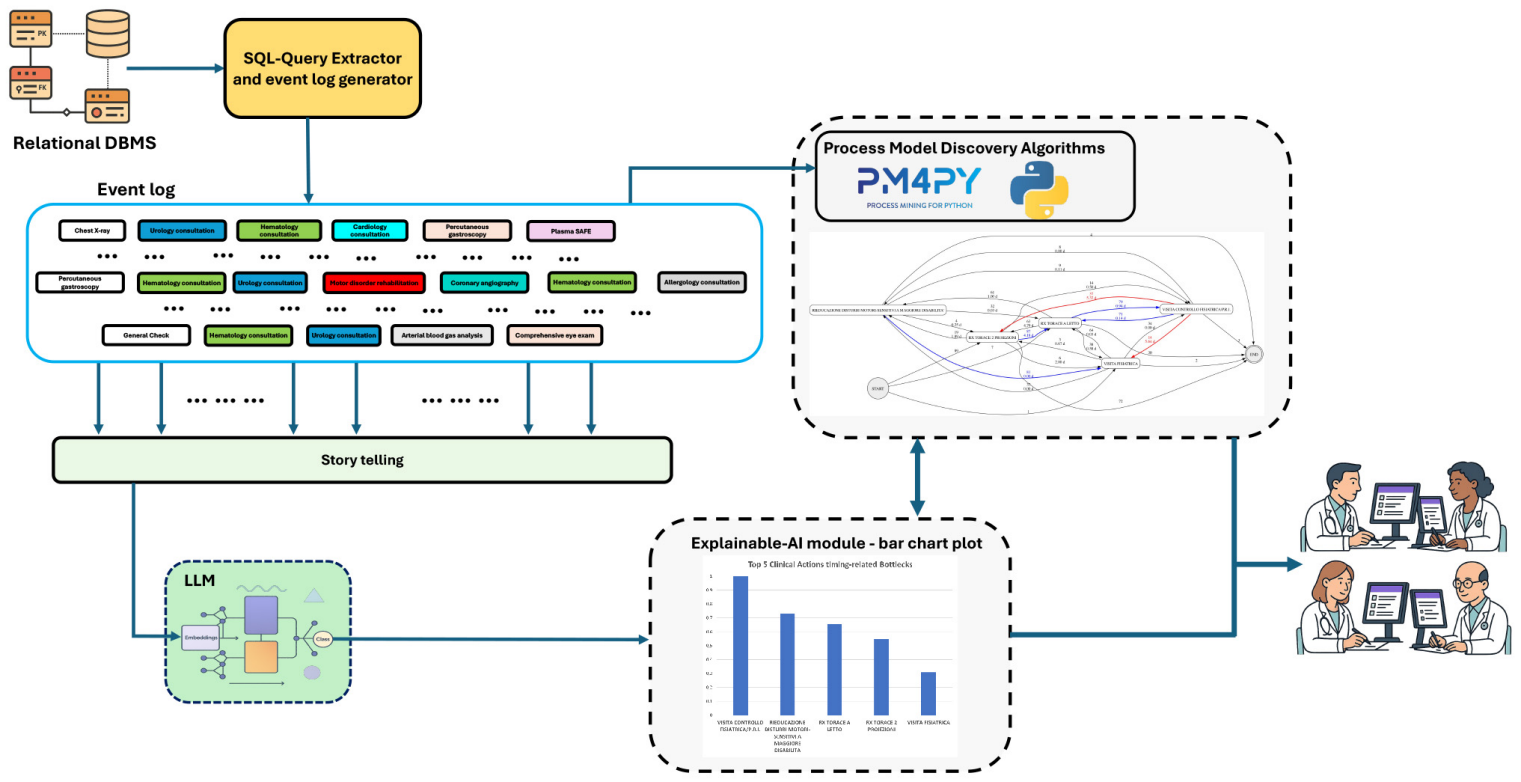
The architecture of TEXLoS.

Figure 1 describes the architecture of TEXLoS. The tool operates according to the following steps:

- Extract the event log from the hospital DBMS. Use the SQL-Query extractor to retrieve OS visits/procedures and generate a time-ordered event log, with a trace for every admitted patient.
- Encode event log traces as model inputs. Encode the event log by story telling: create a narrative description of each patient trace, incorporating both the sequence of OS activities, and their timestamps.
- Classify the traces through an LLM. Feed the embedded traces to a pre-trained LLM (currently, the Transformer-based model BERT; Devlin et al., 2019), to predict the class (class 0 → *LOS* < 20 days, class 1 → *LOS*≥20 days).
- Derive explanations. Calculate Integrated Gradients (IG) to identify which activities most influence each class.
- Discover process structure and timings. Run process model discovery on the same event log [currently, by using Directly Follows visual Miner (DFvM)], highlighting frequency of resource access and time-based bottlenecks.
- Visualize results. Produce interpretable figures (ranked IG bar charts and annotated process models) to explain the results to end users.

Inspired by LEGOLAS (Pasquadibisceglie et al., 2025), in our work we use a LLM to classify OS traces. Currently, we are relying on the Transformer-based Encoder-only model BERT (Devlin et al., 2019) in its base version; BERT is pre-trained, and we fine-tuned it on the 85% of our traces (training set). In particular, we performed a fivefold cross validation on the training set, and then tested the best model on the remaining 15% data. Detailed results are available in the following section.^4^ In order to feed the LLM, we adopt a story-telling approach as a means to provide the deep learning architecture with all the available trace information. Below we show, as an example, an excerpt of a trace (in XES format^5^), and the corresponding story.


<log xes.version="1.0" xes.features="nested-attributes"
   xmlns="http://www.xes-standard.org/">
   <extension name="Concept" prefix="concept" uri="http://www.xes-standard.org/concept.xesext"/>
   <extension name="Time" prefix="time" uri="http://www.xes-standard.org/time.xesext"/>
   <extension name="Lifecycle" prefix="lifecycle" uri="http://www.xes-standard.org/lifecycle.xesext"/>
  
<trace>
   <string key="concept:name" value="5923672"/>
  
   <!-- Cardiology visit: 1 hour duration -->
   <event>
    <string key="concept:name" value="Cardiology visit"/>
<string key="lifecycle:transition" value="start"/>
    <date key="time:timestamp" value="2023-02-06T09:06:00.000+01:00"/>
</event>
  
<event>
    <string key="concept:name" value="Cardiology visit"/>
    <string key="lifecycle:transition" value="complete"/>
    <date key="time:timestamp" value="2023-02-06T10:06:00.000+01:00"/>
</event>



<!-- Diabetology visit: starts 20 minutes after Cardiology completes -->
<event>
   <string key="concept:name" value="Diabetology visit"/>
   <string key="lifecycle:transition" value="start"/>
   <date key="time:timestamp" value="2023-02-06T10:26:00.000+01:00"/>
</event>
<!-- Assumption: Diabetology duration = 10 minutes -->
<event>
   <string key="concept:name" value="Diabetology visit"/>
   <string key="lifecycle:transition" value="complete"/>
   <date key="time:timestamp" value="2023-02-06T10:36:00.000+01:00"/>
</event>
</trace>
</log>



{
"case_id": "5923672",
"story_text": "The patient, 82 years old,
underwent a series of examinations and
interventions during hospitalization. A
Cardiology visit started on February 6th 2023
at 09:06:00 and was completed in 1 hour.
Twenty minutes later, at 10:26:00, a
Diabetology visit started, and was completed
in 10 minutes.
}


As it can be observed, the story is much simpler and compact than the corresponding XES trace. At the same time, narrative encoding allows us to feed the LLM with a semantically richer and more complete input with respect to the sequence of activities/timestamps. In particular, we are allowed to easily provide the LLM with additional activity information or patient data (which may be encoded as activity parameters or additional attributes in the XES document). Indeed, such additional contextual information may remain hidden in aspecific XES tags (such as note fields), and their provision to the model, if a pure “activity sequence + timestamps" format is adopted, would require a complex preprocessing. The choice of relying on stories allows us to make such information explicit, preserving the overall semantics in a very natural way.

BERT then produces an embedding for the input story, and a classification head generates the output.

We thus provide the classification of OS patient traces through the LLM, and make it explainable through the provision of a ranked bar chart which reports the importance (calculated through the IG technique) of the different OS activities in determining the LOS class.

Moreover, the end user can gain additional insight about the nature of the identified bottleneck activities, through process model discovery, learning whether an activity implies a long waiting time before being executed, and/or it is critical because it is applied to a large number of patients. An example of our explainable output is provided in section 4.^6^

## 4 Results

We conducted our experiments on 7,393 real OS traces, collected at *Azienda Ospedaliera SS. Antonio e Biagio e Cesare Arrigo* in Alessandria, Italy, over the years 2022-2023. Some statistics on our event log dataset reveal that the average trace lasted 11.1 days, with a standard deviation of 9.01 days, and 12 OS activities on average (ranging between 2 and 236).

We aimed at distinguishing between ≥20 days and < 20 days LOS. The 20-day cutoff for defining “short" vs. “long," has been set in accordance with domain experts. The dataset is imbalanced, with 89.06% of the traces in class 0 (LOS < 20 days) and 10.94% in class 1 (LOS ≥ 20 days). We thus applied an oversampling technique on the training set (85% of the available data), relying on SMOTE (Chawla et al., 2002), augmenting class 1 up to 2,427 items.

As already mentioned, we performed a fivefold cross validation on the training set, and then tested the best model on the 15% test set. In order to remove possible biases, all folds and the test set were stratified with respect to the patient diagnosis and ward. As regards oversampling, cross validation was realized guaranteeing that the split always maintained the class proportion in the fivefolds.

The loss function we applied is the focal loss (Lin et al., 2017), particularly suited for highly imbalanced data. Indeed, it addresses class imbalance by reshaping the standard cross entropy loss such that it down-weights the loss assigned to the examples that are well classified. Additional hyperparameters are as follows: max token length: 512; optimizer: ADAMW; batch size: 48; learning rate: 5e-06; number of epochs: 20; early stopping: true; patience: 5. On a AMD Ryzen 9 5900X processor equipped with 64GB RAM and Nvidia RTX 4090 GPU, BERT fine tuning took 5 min and 42 s, while story construction took 1 min.

Table 1 presents our classification results on the test set. In the table, we compare TEXLoS to two simpler approaches. The first one relies on a Long Short Term Memory (LSTM) network, structured as follows: a first LSTM layer with 256 units with a *tanh* activation function is followed by a second LSTM layer that consists of 128 units with *tanh* activation; then, a dropout layer with a 0.5 rate helps prevent overfitting by randomly deactivating half of the neurons during training. After such layers, a dense layer with 16 units and a *relu* activation function is applied to learn complex relationships and reduce dimensionality. Finally, a classification layer with a *sigmoid* activation function outputs a probability score for binary classification. The second simpler approach, in an ablation study perspective, relies on BERT, but does not resort to story-telling: BERT is directly fed with the sequence of trace activities, concatenated with their temporal information. The higher performance of TEXLoS (results are highlighted in bold in the table) justifies the choice of a more articulated approach: in particular, a balanced accuracy of 93% was reached, along with a Matthews Correlation Coefficient (MCC) of 0.68. Moreover, and more importantly, the choice of relying on a Transformer-based architecture, and on the story-telling approach, naturally moves a significant step toward explainability, thanks both to natural language input provision, which is semantically richer, and to a more easily interpretable output, as we will showcase below. The confusion matrix on the test set for TEXLoS is also shown in Table 2. For the sake of completeness, it is worth adding that the average balanced accuracy in cross validation was 90%, with a variance of 0.02.

**Table 1:** Top: results obtained by TEXLoS, compared to simpler approaches (LSTM and BERT applied to traces (BERT-TS)).

Approach	Class	Precision	Recall	F1-score	Specificity	MCC	K-Stat	Accuracy
**TEXLoS**	< 20 days (class 0)	0.98	0.93	0.96				
**TEXLoS**	≥20 days (class 1)	0.60	0.86	0.71				
**TEXLoS**	**Weighted / Overall**	**0.94**	**0.93**	**0.93**	**0.86**	**0.68**	**0.67**	**0.93**
**LSTM**	< 20 days (class 0)	0.91	0.90	0.91	0.78			
**LSTM**	≥20 days (class 1)	0.75	0.78	0.76	0.90			
**LSTM**	**Weighted / Overall**	0.87	0.87	0.87	0.81	0.67	0.67	0.87
**BERT-TS**	< 20 days (class 0)	0.98	0.93	0.95	0.82			
**BERT-TS**	≥20 days (class 1)	0.57	0.82	0.67	0.93			
**BERT-TS**	**Weighted / Overall**	0.93	0.91	0.92	0.83	0.64	0.63	0.91

**Table 2:** Bottom: confusion matrix obtained by the TEXLoS.

Actual ↓	Class 0	Class 1	←Predicted
< 20 days (class 0)	920	68	
≥20 days (class 1)	17	104	

↓ indicates the actual class, while ← indicates the predicted class.

**Figure 2:**
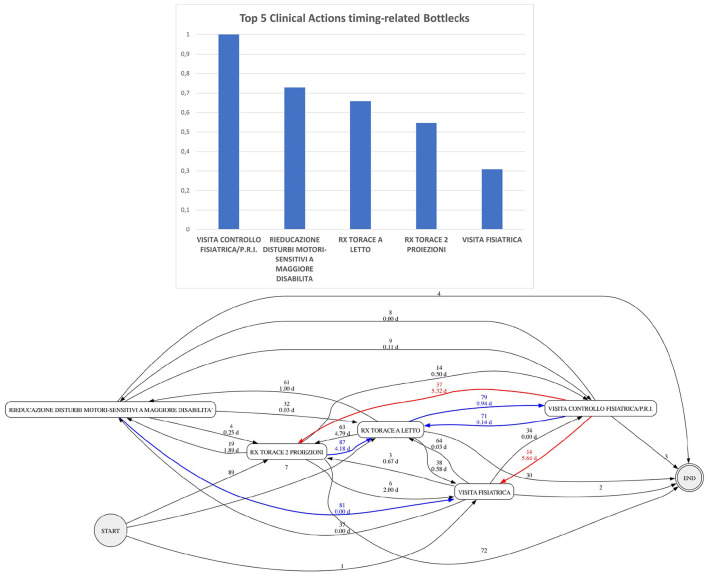
TEXLoS output for end users in the cardio-surgery ward use case. **Top**: bar chart plot showing the most impacting OS activities on LOS (Translation from Italian: “Physiatric Check/(Re)-Definition of Personalized Rehabilitation Plan”; “Motion and Sensing Disability Re-education Session”; “Chest X-rays in Bed”; “Chest X-rays in 2 projections”; “Physiatric visit”). **Bottom**: process model highlighting temporal bottlencks (in red) and frequency bottlenecks (in blue).

As a concrete example to discuss explainability, we will show the use case of a hospital administrator, who exploits TEXLoS to inspect the impact of OS activities on LOS, focusing on a group of about 200 patients, admitted at the cardio-surgery ward for an intervention on heart valves. Figure 2 (top) shows a bar chart, presenting the five most impacting OS activities on such a cohort, generated by applying IG on the LLM-based classifier output. As it can be observed they include three specialistic consultations in the area of motion rehabilitation (a physiatric visit, a physiatric check to (re)-defined the personalized rehabilitation plan, and a neuro-motion re-education session), and two radiological examinations (chest X-rays - one of them at the patient's bed).

After having visualized the bar chart plot, the end user is supported by our tool in a further investigation of the possible activity impact on LOS by process model discovery. S/he can mine the process model for the patient subgroup at hand, and visualize the waiting time for the execution of the activities (especially those identified by the bar chart), as well as their frequency. In Figure 2 (bottom), the most critical temporal bottleneck activities are identified by incoming red edges, while frequency bottleneck ones are identified by incoming blue edges. All frequencies and waiting times are reported on the model edges; the thresholds for edge coloring are parameters whose values can be changed. Figure 2 shows that the rehabilitation session is not a bottleneck. The physiatric check for plan (re)-definition, instead, is executed by a high number of patients (blue edge) - indeed, the personalized rehabilitation plan can be changed after some days, and the same patient may undergo this action multiple times - but is temporally efficient. On the other hand, the physiatric visit, which is a more accurate specialistic assessment, is both required by many patients, and temporally inefficient (some patients have to wait for five days or more for being served - red edge). This outcome was expected by the domain experts collaborating with us, as the physiatric personnel resources are relatively limited in the hospital. A less expected outcome is the evidence that chest X-rays are bottenecks as well: in particular, standard chest X-rays may require more than five days of waiting time, and are executed by a very large number of patients (both red and blue edges). Such a bottleneck is hardly acceptable, and suggests the need for a quick evaluation of the radiology workflow, in search for an optimization. Actually, BPM solutions could be seamlessly integrated with TEXLoS to this end - but this topic is outside the scope of the current paper.

It is worth noting that we also generated the IG bar chart on the BERT-TS output (not shown due to space constraints). In that case, however, a nephrological activity was identified in the top five impacting OSs, an outcome that appeared to be not correct, both on the basis of the experts' opinion, and on the basis of the process model (which does not show nephrological procedures as bottleneck). This result further supports the hypothesis that TEXLoS, through the injection of narrative stories into BERT, better captures the contextual semantics of the patient pathway, with respect to BERT-TS, which relies solely on activity sequence and time information.

**Figure 3:**
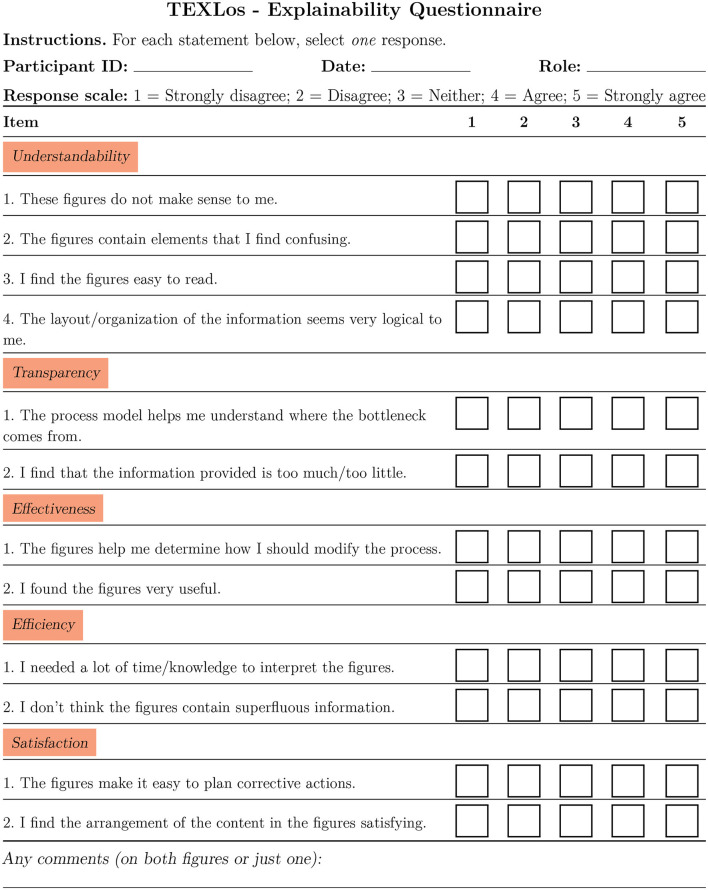
TEXLos - Explainability questionnaire.

We evaluated the perceived quality of the two plots provided to the end users in Figure 2, as a means for explaining the system output, by exploiting a questionnaire. Such a questionnaire, shown in Figure 3, is adapted from (Deters et al. 2025), and collapses in a single form the main dimensions identified in that paper (namely: understandability, transparency, effectiveness, efficiency, and satisfaction). The results were collected on a 1–5 Likert scale.

Note that we reversed the scores of some questions, which are negative statements (question 1 and 2 in the understandability section, question 2 in the transparency section, question 1 in the efficiency section, see Figure 3).

By means of this initial evaluation study, on six end users we obtained an average score of 4/5 (with 0.56 standard deviation) in understandability, 3.66/5 (with 0.47 standard deviation) in transparency, 4.33/5 (with 0.47 standard deviation) in effectiveness, 3.91/5 (with 1.06 standard deviation) in efficiency and 3.58/5 (with 0.11 standard deviation) in satisfaction, leading to a global score of 3.95/5 (with 0.29 standard deviation) indicating, overall, a good perception of our tool as an explainable one. Different process mining visualizations, available in ProM, may be embedded in the future, to increase satisfaction and transparency, by facilitating the identification of issues and the planning of solutions.

## 5 Discussion and conclusions

In this work we have presented TEXLoS, a tool grounded on state of the art techniques, able to analyse patient traces in a post-hoc fashion, in search of OS activities that acted as bottlenecks, and possibly prolonged LOS. Specifically, the very good classification performance we obtained by means of our LLM-based approach suggests that OSs may play a role on prolonging hospitalizations: therefore, organizational issues should be carefully addressed to optimize the service provided to patients and to reduce costs. The identification of the most impacting activities, provided through bar chart plots, and further explained by process models decorated by bottleneck information, can help end users in implementing well-focused planning strategies.

It is important to note that, within the TOT-AL project, which funded our work, we also made other analyses, meant at understanding the reasons for increased LOS. We afforded the problem both in a supervised manner (through classification, considering different patient types, such as those affected by comorbidities or specific pathologies), and in an unsupervised one (through clustering, in order to verify if more implicit factors had a clear correlation with long LOS). Results did not show interesting regularities, therefore we do not think that the current work is affected by strong confounding factors. Moreover, the fact that only a small portion of patients are characterized by a long LOS, across all the wards, does not suggest that LOS is significantly influenced by ward-specific policies. However, as long as we continue to collect new patients data and increase more and more the dataset dimension, we may define additional experiments, to better separate patient types, for example, according to case severity.

It is also worth noting that our approach is based on predictive classification, and is thus able to identify patterns associated with longer LOS, but does not formally establish a causal effect between OSs and LOS itself. In our future work, we will consider strengthening the analysis in the direction of causality, possibly by exploring causal machine learning approaches (see Kaddour et al., 2025) to complement our predictive framework.

Further developments are scheduled. In particular, the choice of adopting a classifier taking in input a narrative textual description of the trace could allow us to feed TEXLoS directly with discharge letters, by-passing the querying phase from the hospital DBMS. Notably, the LLM itself could also translate the discharge letter into a trace (somehow reversing the current workflow steps order, see Bottrighi et al., 2026), thus maintaining the possibility of a seamless integration with process discovery.

Moreover, we acknowledge that our validation study on end users at the *Azienda Ospedaliera SS. Antonio e Biagio e Cesare Arrigo* in Alessandria is still a bit preliminary, since only six individuals answered the questionnaire. However, the encouraging results we obtained so far let us foresee a more systematic deployment of the tool in hospital practice.

## Data Availability

The data analyzed in this study is subject to the following licenses/restrictions: hospitalization data are not publicly available. We generated an exemplary artificial dataset, available in the repository which link is available in the paper. Requests to access these datasets should be directed to stefania.montani@uniupo.it.

## Footnotes

1
https://leemans.ch/visualminer/home/

2
https://fluxicon.com/disco/

3
https://leemans.ch/quickvisualiser/

4 Interestingly, we also made tests on BioBERT and PubMedBERT, which are pre-trained on bio-medical data, but they did not improve classification performance

5
http://www.xes-standard.org/

6 Our code is available at https://osf.io/p64h8/wiki?view_only=d742f30f977d469ba89b707701c2465c&wiki=e8ydm, along with a sample of artificial traces.
